# Oxygénothérapie hyperbare dans la prise en charge du pied diabétique: expérience d’un service de médecine interne à propos de 80 cas

**DOI:** 10.11604/pamj.2018.30.100.14826

**Published:** 2018-06-05

**Authors:** Mohamed Jira, Naoual El Omri, Youssef Sekkach, Fadoua Mekouar, Taoufik Amezyane

**Affiliations:** 1 Service de Médecine Interne B, Hôpital Militaire d’Instruction Mohammed V, Rabat, Maroc

**Keywords:** Oxygénothérapie hyperbare, pied diabétique, médecine interne, Hyperbaric oxygen therapy, diabetic foot, internal medicine

## Abstract

L'oxygénothérapie hyperbare (OHB) est un procédé thérapeutique dans la prise en charge des lésions chroniques menaçant le pied chez les patients diabétiques. Dans le but d'évaluer ce traitement, les auteurs proposent une analyse rétrospective des patients diabétiques présentant une ou plusieurs lésions chroniques aux niveaux des membres inférieurs et ayant bénéficier de l'OHB. Il s'agit de 80 patients, hospitalisés dans un service de médecine interne sur une période de 10 entre janvier 2006 et décembre 2016. Ces patients ont bénéficié de 20 ± 5 séances d'OHB à raison de 2,5 atmosphères absolues sous oxygène pur pendant 90 minutes par séance et par jour associées à une insulinothérapie, une antibiothérapie adaptée, des soins locaux quotidiens. L'évolution des lésions a été évaluée par l'examen clinique avec des mesures répétées de la taille des lésions à la cinquième, quinzième et vingtième séance d'OHB. Notre étude a inclus 80 patients dont 72 hommes et 8 femmes, leur age moyen était de 56,2 ± 4,62 ans, 77,5% présentaient un diabète type 2, évoluant depuis 13,9±3,3 ans. Ils avaient des lésions chroniques du pied (grade 2, 3 ou 4 de Wagner). Une réduction significative de la surface de la plaie a été observée dés la dixième séance avec cicatrisation complète et guérison chez 56 (70%) de nos patients au terme de 20 séances d'OHB. L'amputation a été notée chez 20 (25%) patients. L'OHB est reconnue dans le traitement des lésions chroniques du pied chez le diabétique, elle permet de réduire considérablement l'incidence des amputations.

Hyperbaric Oxygen Therapy (HOT) is a therapeutic procedure for treatment of chronic foot-threatening lesions in diabetic patients. We conducted a retrospective study of patients with diabetes with one or several chronic lower limb lesions having undergone HOT in order to evaluate this treatment. The study involved 80 patients hospitalized in a Department of Internal Medicine during a period of 10 years, between January 2006 and December 2016. These patients underwent 20 ±5 HOT sessions breathing pure oxygen at 2.5 times atmospheric pressure for 90 minutes per session and per day associated with insulin therapy, suitable antibiotic therapy and daily local care. The outcome of lesions was evaluated based on clinical examination by repeated measures of the size of the lesions at the fifth, fifteenth and twentieth HOT session. Our study included 80 patients (72 men and 8 women) with an average age of 56.2 ± 4.62 years; 77.5% had type 2 diabetes, evolving over 13.9±3.3 years. They had chronic foot lesions (Wagner grade 2, 3 or 4). A significant reduction in wound surface area was observed from the tenth session, with complete healing and recovery in 56 (70%) patients at the end of 20 sessions of HOT. Amputation was performed in 20 (25%) patients. HOT is known as a cure for chronic foot lesions in diabetic patients. It can greatly reduce the incidence of amputations.

## Introduction

L'ulcère des extrémités inférieures est estimé à 5-7% de la population diabétique [1-3], il représente un souci majeur en raison du haut risque de complications en menaçant le pied [4,5]. Chez le diabétique, la présence d'un artériopathie des membres inférieurs et d'une neuropathie périphérique prédisposent à ces ulcérations [6,7], dont le mécanisme favorisant est représenté essentiellement par l'infection et l'hypoxie [8-10]. L'oxygénothérapie hyperbare (OHB) est un procédé thérapeutique dans la prise en charge des atteintes ischémiques menaçant le pied chez les patients diabétiques, en raison de son effet antimicrobien en augmentant l'activité bactéricide des leucocytes, formation du tissu par stimulation de la prolifération des fibroblastes et synthèse de collagène, il permet aussi la formation de la microcirculation par réduction d'œdème et par l'angiogénèse [11-14]. Dans le but d'évaluer ce traitement, les auteurs rapportent cette étude en précisant l'intérêt de l'OHB dans les lésions chroniques du pied chez le diabétique.

## Méthodes

Il s'agit d'une étude rétrospective de 80 patients diabétiques présentant une ou plusieurs plaies chroniques aux niveaux des membres inférieurs (mal perforant plantaire, infections ou gangrènes ou phlegmons), colligés dans un service de médecine interne à l'hôpital militaire d'instruction Mohammed V entre Janvier 2006 et Décembre 2016. Ces patients ont bénéficié de 20±5 séances d'OHB (Caisson hyperbare multiplace à raison de 2,5 atmosphères absolues (ATA) sous oxygène pur pendant 90 minutes par séance et par jour) associées à une insulinothérapie, une antibiothérapie adaptée, des soins locaux quotidiens, la mise en décharge du membre atteint quelques fois un geste chirurgical. L'évolution des lésions a été évaluée par l'examen clinique avec des mesures répétées de la taille des lésions à la cinquième, quinzième et vingtième séance d'OHB.

## Résultats

**Figure 1:**
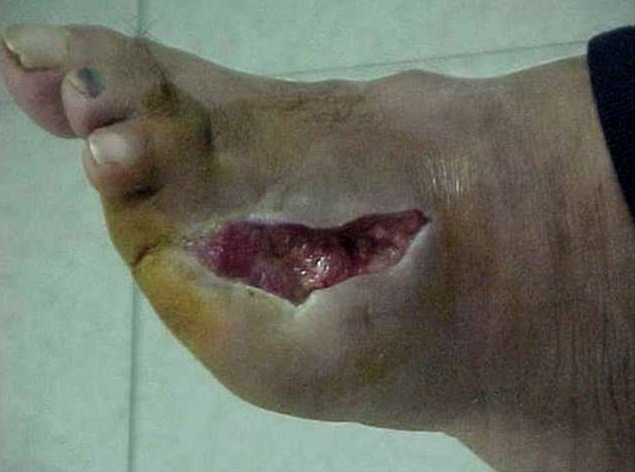
Ulcère profond du pied chez le diabétique avant OHB

**Figure 2:**
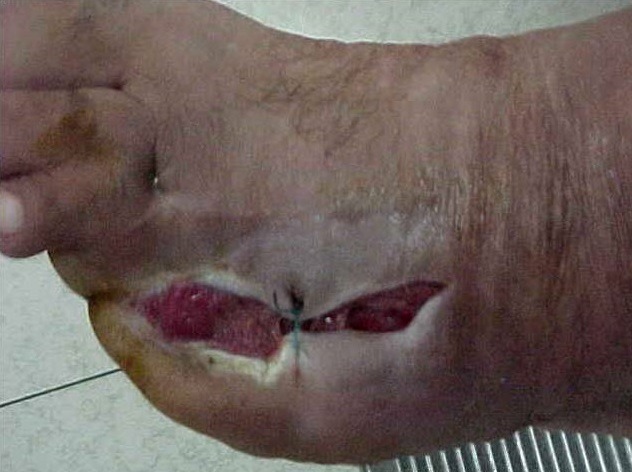
Bourgeonnement d’ulcère, après 10 séances d’OHB

**Figure 3:**
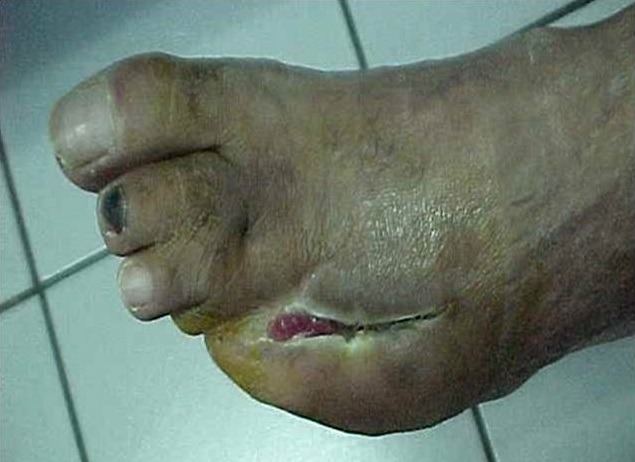
Cicatrisation d’ulcère après 15 séances d’OHB

**Figure 4:**
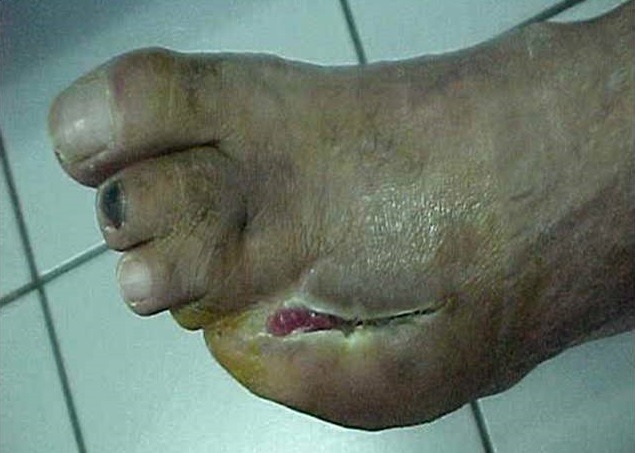
Cicatrisation d’ulcère après 20 séances d’OHB

Parmi nos patients, il s'agissait de 72 hommes et 8 femmes, leur age moyen était de 56,2±4,62 ans (37-78 ans), dont 77,5% présentaient un diabète de type 2, la glycémie à jeun était supérieure à 2g/l et une hémoglobine glycosylée (HbA1c) était supérieure à 7,2. le diabète évoluait depuis 13,9±3,3 ans en moyenne, il était compliqué de neuropathie (90,5%), de rétinopathie (37,5%), de néphropathie (17,5%), d'hypertension artérielle (12,5%) et d'artériopathie des membres inférieurs (15%). Les patients ont été hospitalisés pour des lésions chroniques du pied grade 2, 3 ou 4 de Wagner [15] avec 62,5% de gangrènes, 25% d'abcès profond, 20% d'ostéite, 12,5% d'ulcère profond. Une réduction significative de la surface des lésions a été observée dés la dixième séance avec cicatrisation complète et guérison chez 70% de nos malades au terme de 20 séances d'OHB (Figure 1, Figure 2, Figure 3, Figure 4). L'amputation n'a pu être évitée chez 20 patients (25%) en raison de l'extension de la nécrose tissulaire et de l'infection osseuse favorisée par un état vasculaire précaire et une perturbation métabolique importante. Quatre patients ont du arrêter leur traitement en raison d'un barotraumatisme de l'oreille dans trois cas et d'une tuberculose pulmonaire évolutive chez une patiente.

## Discussion

La nette prédominance masculine observée dans notre série et la littérature [16,17] peut être due aux facteurs de risque cardiovasculaires (alcool, tabac) auxquels l'homme est plus exposé, une protection de la femme par les oestrogènes et au mode de recrutement militaire dans notre contexte. L'âge des patients selon les études est variable entre 60 et 70 ans [18-20]. Le diabète type 2 reste largement le plus fréquent, vu l'âge des patients, l'âge de découverte de diabète et de multiples complications dégénératives [17]. Les diabétiques ayant des lésions du pied stade 2 à 4 selon la classification de Wagner sont candidats à l'OHB, l'HbA1c est supérieur à 7. La majorité des patients avaient des complications dégénératives (néphropathies, rétinopathies et neuropathies) [16,17,19]. L'efficacité de l'OHB sur les lésions du pied reste très difficile à apprécier, du fait de l'absence de groupe témoin des patients traités par des méthodes conventionnelles devant un groupe traité par OHB. Cette étude comparative reste très difficile à réaliser, car le groupe témoin supposerait avoir le même profil épidémiologique, même contrôle métabolique, mêmes états vasculaire et neurologique et le même type de lésion de pied. Chen et al. ont noté dans une étude prospective, randomisée, ouverte et contrôlée, une cicatrisation complète d'ulcère du pied chez 5 (25%) patients du groupe OHB (n=20) contre un (5,5%) patient du groupe traitement conventionnel (n=18), le taux d'amputation était de 5% pour le groupe OHB et de 11% pour le groupe traitement conventionnel [21]. Baroni G et al ont rapporté dans une étude rétrospective non randomisée, 28 patients dont 18 traités par OHB et dix autres formant un groupe contrôle avec des critères similaires de sélection, le groupe OHB a reçu en moyenne 34±2,8 séances de 90 mn/jour à 2,5 ATA d'oxygène pur; la guérison a été observée chez 16 patients de groupe OHB versus un patient de groupe contrôle [22].

Dans une autre étude de 80 patients dont 62 patients ont été mis sous 72±29 séances d'OHB à 2,5 ATA d'oxygène pur et 18 étant un groupe contrôle; Oriani et al. ont rapporté une guérison satisfaisante obtenue chez 59 patients et trois amputations dans le groupe OHB contre 18 amputations chez les patients du groupe contrôle [23]. En 1992 Oriani et al ont rapporté dans une troisième étude que parmi 150 patients traités par OHB (en absence de groupe contrôle), 130 ont été guéri [24]. Dans notre étude la guérison a été obtenue chez 56 patients parmi 80 traités par OHB contre 24 amputations. Faglia et al. Ont rapporté dans une étude prospective randomisée de 68 patients, 8,6% amputations dans le groupe de patients traités par OHB versus 33,3% dans le groupe de contrôle et chez les patients qui avaient des lésions stade IV de Wagner (gangrènes des orteils et pieds), l'amputation a été notée chez 9,1% (2 parmi 22 patients) dans le groupe OHB contre 55 % (11 parmi 20 patients) dans le groupe de contrôle [25]. Dans une autre étude de 115 patients, réalisée par Faglia et al. en 1998, l'OHB a permet une réduction significative des amputations [26]. En 2002, dans une étude prospective de 37 patients diabétiques et présentant un ulcère chronique du pied dont 17 patients ont été mis sous 40 à 60 séances d'OHB et 21 patients ont été traité par les méthodes conventionnelles (La tension artérielle périphérique, les HbA1c, la durée de diabète et les valeurs de la pression transcutanée de l'oxygène étaient semblables dans les deux groupes). La guérison avec une cicatrisation complète a été obtenue chez 76 % des patients du groupe OHB versus 48% du groupe contrôle, la durée de suivi était de trois ans. L'amputation a été notée chez deux patients (12%) du groupe OHB contre sept patients (33%) de groupe contrôle [19]. Kessler et al ont rapporté une réduction importante de la taille de l'ulcère dans le groupe OHB (41.8±25.5% contre 21.7±16.9% dans le groupe de contrôle), après 15 jours d'OHB [17]. Selon ces études, l'OHB a son intérêt dans le traitement des lésions chroniques du pied chez le diabétique, permettant d'éviter l'amputation.

## Conclusion

L'OHB constitue un progrès thérapeutique réel dans la prise en charge des lésions du pied diabétique, elle est efficace dans les cas difficiles. Des indications posées précocement pourraient accélérer l'obtention de bons résultats.

### Etat des connaissances actuelle sur le sujet

- Intérêt de l'OHB dans le traitement des lésions chroniques du pied chez le diabétique.

### Contribution de notre étude à la connaissance

- Utilisation précoce d'OHB permet d'éviter les amputations du pied chez le diabétique.

## Conflits d’intérêts

Les auteurs ne déclarent aucun conflit d'intérêts.
